# Novel Modulators of the Growth Hormone - Insulin-Like Growth Factor Axis: Pregnancy-Associated Plasma Protein-A2 and Stanniocalcin-2

**DOI:** 10.4274/jcrpe.2017.S001

**Published:** 2017-12-30

**Authors:** Masanobu Fujimoto, Vivian Hwa, Andrew Dauber

**Affiliations:** 1 Cincinnati Children’s Hospital Medical Center, Cincinnati Center for Growth Disorders, Clinic of Endocrinology, Cincinnati, Ohio, USA

**Keywords:** Pregnancy-associated plasma protein-A2, stanniocalcin-2, insulin-like growth factor-1, growth hormone

## Abstract

Growth hormone (GH) and its mediator, insulin-like growth factor-1 (IGF-1), play a critical role in human growth. In circulation, IGF-1 is found in a ternary complex with IGF binding proteins (IGFBPs) and acid labile subunit (ALS) but little attention has been paid to the regulation of IGF-1 bioavailability. Recently, pregnancy-associated plasma protein-A2 (PAPP-A2) and stanniocalcin-2 (STC2) were identified as novel modulators of IGF-I bioavailability. PAPP-A2 is a protease which cleaves IGFBP-3 and -5, while STC2 inhibits PAPP-A and PAPP-A2 activity. In collaboration with a group in Madrid, we reported the first human cases carrying mutations in the PAPPA2 gene who presented with short stature, elevated total IGF-1, IGFBP-3, IGFBP-5 and ALS, but low free IGF-1. Additionally, the patients demonstrated insulin resistance and below average bone mineral density (BMD). The PAPP-A2 deficient patients were treated with recombinant human IGF-1, resulting in improvements in growth velocity, insulin resistance, and BMD. These findings suggested that the bioactive, free IGF-1 liberated from IGFBPs by PAPP-A2 is important for human growth. Mouse models of PAPP-A2 and STC2 provide further insights into their roles in growth physiology. This review will summarize new insights into PAPP-A2 and STC2 and their role in the GH-IGF axis, thereby highlighting the importance of the regulation of IGF-1 bioavailability in human health and disease.

## INTRODUCTION

**Figure 1:**
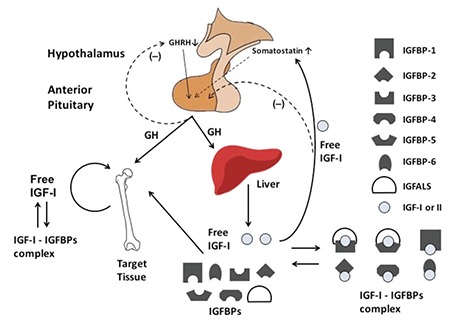
Schematic of the growth hormone - insulin-like growth factor-1 axis in human growth. Growth hormone secretion produces insulin-like growth factor-1 in the liver and at the local target tissue, such as the growth plate. Growth hormone also regulates the expression of insulin-like growth factor binding proteins and insulin-like growth factor acid labile subunit from the liver. Insulin-like growth factor-1 circulates bound to insulin-like growth factor binding proteins and insulin-like growth factor acid labile subunit in serum. Free insulin-like growth factor-1 liberated from insulin-like growth factor binding proteins is the active form of the hormone IGF-1: insulin-like growth factor-1, IGFBP: insulin-like growth factor binding protein, GH: growth hormone, GHRH: growth hormone-releasing hormone, IGFALS: insulin-like growth factor acid labile subunit

Short stature is a very common complaint usually seen by pediatric endocrinologists. The growth hormone (GH) - insulin-like growth factor 1 (IGF-1) axis plays a central role in childhood growth (Figure 1). Human genetic defects affecting this axis lead to a variety of growth disorders (1) and have provided a wealth of knowledge about growth biology. Recently, human genetic studies have pointed to the importance of new components of this axis affecting the regulation of IGF-1 bioavailability. In this article, we will focus on two genes which play critical roles in regulating IGF-1 bioavailability, pregnancy-associated plasma protein-A2 (PAPP-A2) and stanniocalcin-2 (STC2). We will review the two genes followed by lessons learned from genome-wide association (GWA) studies of adult height in the general population. We will then discuss the recently discovered human mutations in PAPP-A2 and conclude with a brief review of what has been learned from animal models of these two genes.

### Novel Members of the Growth Hormone - Insulin-like Growth Factor System - PAPP-A2 and STC2

**Figure 2:**
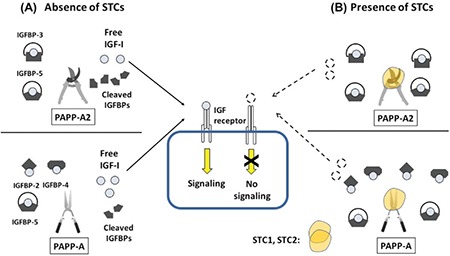
The action of pregnancy-associated plasma protein-A, -A2, and stanniocalcin-1 and -2 on insulin-like growth factor binding proteins in insulin-like growth factor signaling. A) Pregnancy-associated plasma protein-A2 and pregnancy-associated plasma protein-A action without the presence of stanniocalcins. Pregnancy-associated plasma protein-A2 can cleave insulin-like growth factor binding protein-3 and -5 and pregnancy-associated plasma protein-A can cleave insulin-like growth factor binding protein-2, -4, and, -5, resulting in liberation of free insulin-like growth factor-1. Because free insulin-like growth factor-1 can bind its receptor, insulin-like growth factor-1 signaling is then induced. B) Pregnancy-associated plasma protein-A2 and pregnancy-associated plasma protein-A action in the presence of stanniocalcins. Stanniocalcins inhibit pregnancy-associated plasma protein-A2 and -A’s ability to cleave insulin-like growth factor binding proteins thereby resulting in decreased levels of free insulin-like growth factor-1 and consequently decreased insulin-like growth factor-1 signaling IGF: insulin-like growth factor, IGFBP: insulin-like growth factor binding protein, STC: stanniocalcin, PAPP-A: pregnancy-associated plasma protein-A

The PAPP-A2 gene (chromosome 1q25.2) encodes the pregnancy-associated plasma protein-A2, a member of the pappalysin family of metzincin metalloproteinases. PAPP-A2 cleaves IGF binding proteins 3 and 5 (IGFBP-3 and IGFBP-5) thereby liberating IGF-1 from its ternary complex which leads to increased, bioactive, free IGF-1 (Figure 2) (2,3). PAPP-A2 protein is widely expressed in human tissues, especially in the placenta and is detected at high levels in the circulation of pregnant women during the first trimester and at term (4). PAPP-A2 is 46% homologous with the closely related protein PAPP-A.

The STC family of proteins has two members, STC1 and STC2, both of which are highly conserved from fish to higher vertebrates (5). The STC2 gene is located on chromosome 5 (5q35.2) and is a widely expressed, secreted homo-dimeric glycoprotein (6). Given STC1’s role in calcium and phosphate metabolism, STC2 was first investigated for its putative action on phosphate metabolism and cancer metastasis (7,8,9), but it was later found that STC2’s main role is as a component of GH-IGF axis. STC2 was found to be a potent inhibitor of both PAPP-A and PAPP-A2 (10,11) and functions by binding with PAPP-A and PAPP-A2 resulting in their inactivation (Figure 2) (10,11).

### Evidence from Genome-wide Association Studies

Over the past decade, there have been numerous GWA studies (GWAS) examining the role of common genetic variants in determining disease risk, as well as variation in anthropometric traits such as height and obesity. In 2010, the Genetic Investigation of Anthropometric Traits Consortium performed a GWAS of adult height in 183.727 individuals (12). They found 180 different genomic loci associated with stature. While the genetic variant in these loci only explained approximately 10% of the phenotypic variation in height, a closer evaluation of biological pathways implicated by these loci provided insights into growth biology. For example, a number of genes known to play a role in growth such as the GH1 gene as well as genes involved in transforming growth factor-β signaling and the growth plate matrix were identified. Interestingly, additional new genes not previously known to be linked to height were highlighted. For the purposes of this review, it is key to note that both STC2, PAPP-A2, and its related gene PAPP-A were identified as being within genome-wide significant loci. While these three genes had previously been linked to the GH-IGF-1 axis, this was the first time that genetic variation in these genes was linked to human height (12).

**Figure 3:**
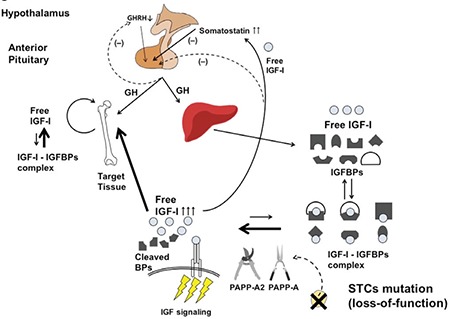
Schematic of the predicted pathophysiology of stanniocalcin-2 deficiency. Mutations which decrease stanniocalcin-2 activity result in decreased inhibition of pregnancy-associated plasma protein-A and pregnancy-associated plasma protein-A2. Therefore, increased protease activities of pregnancy-associated plasma protein-A and -A2 against insulin-like growth factor binding proteins would increase the availability of free bioactive insulin-like growth factor-1. This would be predicted to lead to increased insulin-like growth factor-1 signaling and taller stature IGF-1: insulin-like growth factor-1, GH: growth hormone, GHRH: growth hormone-releasing hormone, BPs: binding proteins, IGFBPs: insulin-like growth factor binding proteins, PAPP-A: pregnancy-associated plasma protein-A, STCs: stanniocalcins

In a follow up GWAS, the effects of rare and low frequency coding variants on human height were investigated, as opposed to the previously studied common (allele frequency >5%) non-coding variants. Eighty-three rare and low-frequency coding variants were found to be associated with human height at a genome-wide significant level. Of these 83 variants, the variant with the largest effect size was found in STC2. The heights of carriers with this rare STC2 gene missense variant were approximately 2.1 cm taller than non-carriers. Functional characterization of the STC2 variant demonstrated that its presence leads to decreased binding of STC2 to PAPP-A in vitro, resulting in decreased inhibition of PAPP-A activity and increased cleavage of IGFBP-4 (Figure 3) (13). Presumably, this would result in increased levels of free IGF-1 although this was not directly investigated. This study provides conclusive evidence linking rare damaging variants in STC2 with increased human height.

### Rare Mutations in Pregnancy-Associated Plasma Protein-A2 Lead to a Novel Growth Disorder

In 2016, our group, in collaboration with Professor Jesús Argente and his colleagues, reported the first two families with rare damaging mutations in PAPPA2 (14). We performed whole-exome sequencing in two families of Spanish and Palestinian ancestry whose children presented with short stature and markedly elevated IGF-1 levels. The families were found to be homozygous for the p.D643fs25 and p.A1033V variants in PAPPA2 respectively (14). Functional studies demonstrated absent expression of the p.D643fs25 mutant at the protein level and significantly reduced expression of the p.A1033V mutant. Importantly, the PAPPA2 p.A1033V mutant was unable to cleave IGFBP-3 and IGFBP-5 confirming the loss-of-function effect of this mutation.

**Figure 4:**
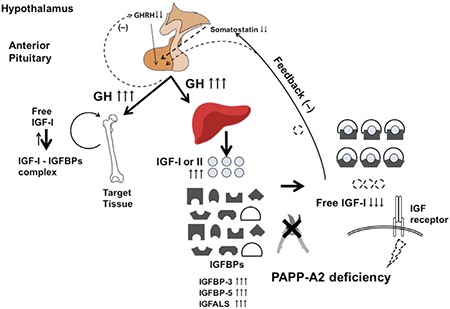
Schematic of the pathophysiology of loss of pregnancy-associated plasma protein-A2 activity. The decreased or mutated pregnancy-associated plasma protein-A2 cannot proteolyze insulin-like growth factor binding protein-3 and -5 resulting in decreased free insulin-like growth factor-1. The reduction of free insulin-like growth factor-1 leads to increased growth hormone secretion due to a lack of negative feedback. Elevated growth hormone levels result in increased production of insulin-like growth factor-1 and -2 and insulin-like growth factor binding proteins. Despite elevation of these hormones, insulin-like growth factor-1 signaling is decreased due to the low levels of free insulin-like growth factor-1 GH: growth hormone, IGF: insulin-like growth factor, IGFBP: insulin-like growth factor binding protein, GHRH: growth hormone-releasing hormone, PAPP-A2: pregnancy-associated plasma protein-A2

The Palestinian family had three affected children with significant short stature (height range -2.8 to -3.8 standard deviation scores) while the two affected Spanish children had short stature relative to their mid-parental target height (14). Based on the growth profile of the one post-pubertal patient, it appears that growth failure is progressive and there is no significant pubertal growth spurt. Two of the five affected patients were born mildly small for gestational age. The parents of both families who were heterozygous for the PAPPA2 mutations were of normal stature. Some of the patients with the homozygous PAPPA2 mutations also presented with moderate microcephaly, small chin, long thin bones, decreased bone mineral density (BMD) and delayed dental eruption. Biochemically, they had elevated total IGF-1, IGFBP-3, IGFBP-5, acid labile subunit and IGF-2 levels, most of which are GH-dependent factors. The bioactive and free IGF-1 levels were either frankly low or in the low-normal range with a marked decrease in the bioactive/total IGF-1 ratio. GH secretion was elevated in the patients. Presumably, PAPP-A2 dysfunction leads to decreased free IGF-1 levels thus resulting in increased circulating GH concentrations due to a lack of negative feedback on GH secretion (Figure 4).

Given the decreased levels of free IGF-1 present in these patients, it was hypothesized that treatment with recombinant human IGF-1 (rhIGF-1) could potentially be beneficial for these patients. This approach was first reported by Muñoz-Calvo et al (15) for the two Spanish patients carrying mutant PAPPA2 p.D643fs25. The rhIGF-1 treatment was administered at a dose of 40-80 mg/kg twice daily for six months. Subsequently, the dose was gradually increased to 120 mg/kg for a total treatment period of one year. The treatment was started at ages 10.5 and six years of age, respectively. Both siblings increased their height by 0.4 standard deviation (SD). Of note, the older girl also received gonadotropin-releasing hormone analog therapy to suppress pubertal development as she entered puberty six months into treatment. Her height velocity increased from 3.7 cm/year (-1.5 SD) pre-treatment to 7.6 cm/year (+1.6 SD) on rhIGF-1 treatment. The younger brother’s height velocity also increased from 5.8 cm/year (-1.6 SD) pre-treatment to 7 cm/year (+1.1 SD) on rhIGF-1 treatment (15). For the Palestinian family, the two younger patients carrying the PAPPA2 p.A1033V mutation were treated with 120 mg/kg of rhIGF-1 (16). The youngest sibling’s height increased by 0.4 SD over a period of one year with a doubling of his height velocity from 3 cm/year pre-treatment to 6.2 cm/year on treatment. Unfortunately, the older brother developed severe headaches caused by increased intracranial hypertension, presumably due to the rhIGF-1 treatment, leading to the discontinuation of therapy. After stopping the rhIGF-1 treatment, his symptoms completely resolved (16). His height SD declined from -2.9 to -3.0 over the year despite progressing in pubertal development.

In addition to the effects on height, the subjects have been investigated for the effects of PAPP-A2 deficiency on metabolic parameters and bone health. The three Palestinian children underwent oral glucose tolerance testing and were found to have significant insulin resistance and pre-diabetes. Interestingly, the youngest sibling had complete resolution of the insulin resistance after one year of treatment with rhIGF-1. One possible explanation is that the medication increased free IGF-1 levels thus resulting in lower GH levels with a consequent decrease in insulin resistance. The three affected individuals also had below average BMD with the youngest sibling having an increase in BMD in response to rhIGF-1 therapy.

### Characteristics of Pregnancy-associated Plasma Protein-A, Pregnancy-Associated Plasma Protein-A2, Stanniocalcin-1, and Stanniocalcin-2 in Mouse Models

Numerous studies have been performed using knock-out (KO) and transgenic (Tg) mouse models to understand the physiology of PAPP-A, PAPP-A2, STC1, and STC2. Many of the findings seen in these in vivo mouse models mimic the features observed in the patients with PAPP-A2 mutations and deepen our understanding of the role that this family of genes play in growth biology. We summarize the phenotypic characteristics of these mouse models in growth, biochemistry, glucose metabolism and bone development in Table 1.

As a first step in understanding the roles of PAPP-A, PAPP-A2, STC1, and STC2 in regulating growth and the GH-IGF axis, anthropometric data of generated mouse models were examined. Homozygous Pappa, Pappa2, Stc1, and Stc2 KO mice are all viable. In contrast, human STC2 (hSTC2) Tg mice in which hSTC2 was overexpressed had decreased viability with 26-34% neonatal mortality without apparent dysmorphology (17). Homozygous PAPP-A KO as well as hSTC1 and hSTC2 Tg over-expression mice showed approximately a 30-40% reduction in birth weight relative to wild-type (WT) mice (6,17,18). All three of these models should lead to decreased IGF-1, and possibly IGF-2, bioavailability which is consistent with the decreased birth size. At the other end of the spectrum, homozygous Stc2 KO mice, which should have increased IGF-1 bioavailability, were born with a birth weight that was 15% heavier than WT (5). Interestingly, there was no significant difference in birth weight between homozygous Pappa2 or Stc2 KO mice and WT (19,20). As noted above, only two of the five patients with PAPPA2 mutations were born small for gestational age, suggesting that perhaps there is a mild effect of PAPPA2 on in utero growth in humans that was not present in the current mouse model. Furthermore, the growth limiting (Pappa KO, Pappa2 KO, STC1 and STC2 overexpression) and growth promoting (Stc2 KO) effects of all KO and Tg mice persisted or became more apparent in post-natal growth (Table 1). Stc1 KO mice remained the same size as WT mice throughout their lives suggesting that STC1 plays a less important role in growth physiology. These results hint at the possibility that these genes could have differential roles in pre- and post-natal growth.

Total IGF-1 values, but not free bioactive IGF-1 were measured in all of the mouse models. Consistent with the human PAPPA2 mutation patients, total IGF-1 values were higher than WT in homozygous Pappa2 KO and male hSTC2 Tg mice but not female hSTC2 Tg mice (6,21). In the remaining mouse models, there were no differences in total IGF-1 between mutants and WT (5,17,18). Free IGF-1 values which were measured in the Pappa2 KO mice were decreased (14). In future studies, it will be important to measure free IGF-1 levels in the other mouse models. There is limited additional data regarding the other biochemical marker of the GH-IGF axis, such as GH and IGFBPs (Table 1). The homozygous Pappa2 KO animals had increased IGFBP-5 levels and variable levels of IGFBP-3 compared with WT (21,22). There was no difference in IGFBP-2 and -4 when these mice were compared with WT (22).

It is well known that IGF-1 has insulin-like activity acting via the insulin receptor and hybrid insulin/IGF-1 receptors. However, there is little data about glucose metabolism in the KO and Tg mice. In homozygous Pappa2 KO mice, the blood glucose levels at baseline and during an intraperitoneal glucose tolerance test did not differ from WT mice (21).

Previous studies have shown that IGF-1, as well as IGFBPs, play a critical role in skeletal growth and maintenance (25,26). Similar to what was seen in the PAPPA2 mutation patients, Pappa and Pappa2 KO mice showed delayed bone development with regards to bone formation and/or mineralization (Table 1). Interestingly, hSTC1 and hSTC2 Tg mice had severe impairment in post-natal, linear axial skeletal growth (24). No significant effects were seen in the Stc1 and Stc2 KO mice.

## Conclusion

Since the year 2000, PAPP-A, PAPP-A2, STC1, and STC2 have been highlighted as new players in regulating IGF-1 bioavailability and thus human growth. Our group previously reported the first PAPP-A2 deficiency cases which had short stature together with abnormal glucose and bone metabolism (14). These patients represent a severe perturbation in the regulation of IGF-1 bioavailability and thus provide insights into the importance of this pathway for growth. Additionally, both common and rare genetic variants in this pathway, found in the general population, have been shown to affect adult height. To date, there have been no reports of human patients with severe STC2 pathogenic mutations. Loss-of-function mutation in STC2 would be expected to cause tall stature while gain-of-function mutations may cause short stature. The KO and Tg mouse models of these genes, as summarized above, are useful tools to probe the fundamental physiology of these novel growth modulators. However, there are still many unanswered questions for future investigations. Finally, there is minimal data about normal levels of PAPP-A2 or STC2 from fetus to adulthood or how their genes’ expression may be regulated. Further studies assessing the roles of PAPP-A2 and STC2 in human growth and bioactive free IGF-1 availability will provide important insights into growth physiology.

## Figures and Tables

**Table 1:**
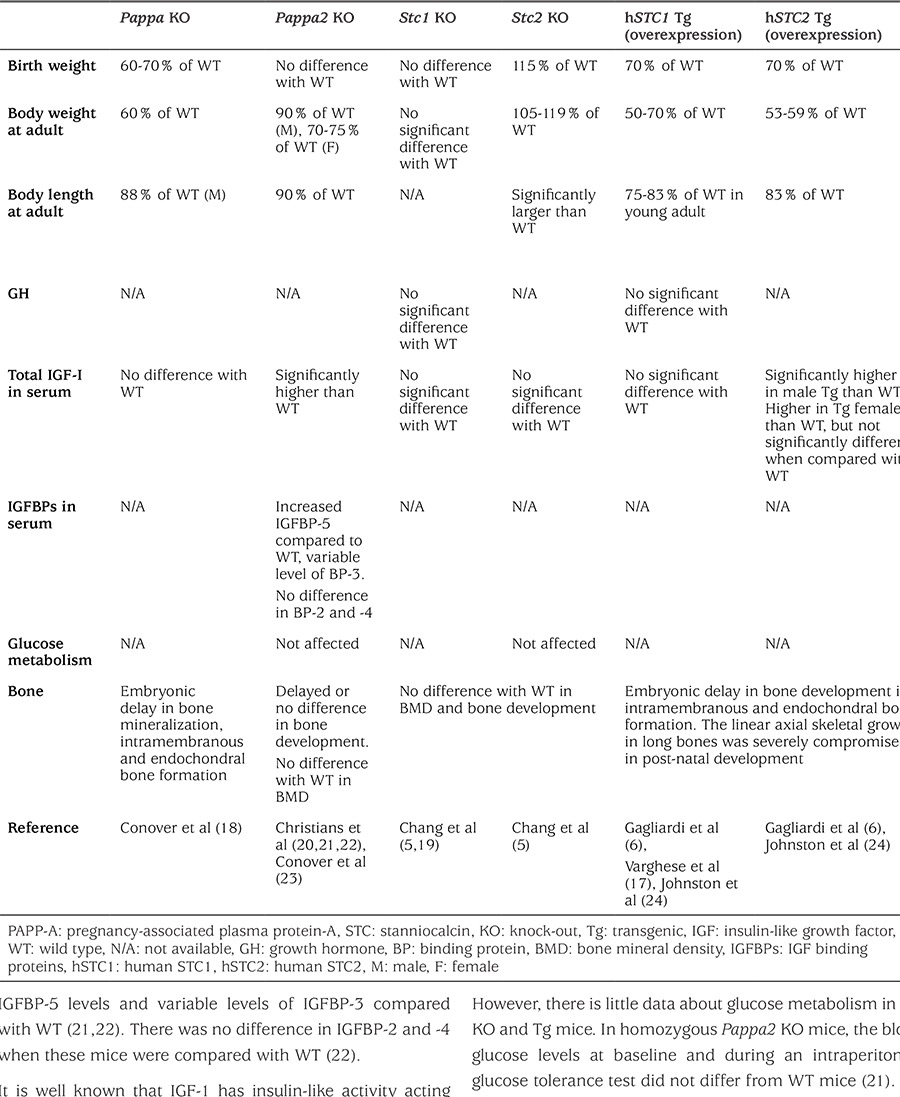
Phenotypic description of pregnancy-associated plasma protein-A, pregnancy-associated plasma protein-A2, stanniocalcin-1, and stanniocalcin-2 mouse models
